# Quantitative analysis of amphiphilic *N*-alkyloxypyridinecarboximidamide by liquid chromatography–tandem mass spectrometry

**DOI:** 10.1007/s11696-016-0019-1

**Published:** 2016-12-10

**Authors:** Irmina Wojciechowska, Aleksandra Wojciechowska, Karolina Wieszczycka, Przemysław Aksamitowski, Joanna Zembrzuska, Grzegorz Framski

**Affiliations:** 1 Institute of Chemical Technology and Engineering, Poznan University of Technology, Berdychowo St. 4, 60-965 Poznan, Poland; 2 Institute of Chemistry and Technical Electrochemistry, Poznan University of Technology, Berdychowo St. 4, 60-965 Poznan, Poland; 3 Institute of Bioorganic Chemistry, Polish Academy of Science, Z. Noskowskiego St. 12/14, 61-704 Poznan, Poland

**Keywords:** *N-alkyloxypyridinecarboximidamide*, *O*-alkylation reaction, LC–MS/MS, Reaction kinetics

## Abstract

LC–MS/MS method to determine hydrophobic *N*-alkyloxy substituted amidines: *N*-(2-ethylhexyloxy)pyridine-2-carboximidamide, *N*-(2-ethylhexyloxy)pyridine-3-carboximidamide, *N*-(2-ethylhexyloxy)pyridine-4-carboximidamide, *N*-decyloxy pyridine-2-carboximidamide, *N*-decyloxypyridine-3-carboximidamide and *N*-decyloxypyridine-4-carboximidamide was developed and validated in terms of linearity, precision and accuracy. The developed method was successfully applied to monitor and control the synthesis process. The experimental data points indicated that the straight chain alkyl bromide reacted most rapidly than branched alkyl bromide and the enhancement of the reaction efficiency strongly depended on reaction temperature.

## Introduction

**Fig. 1:**
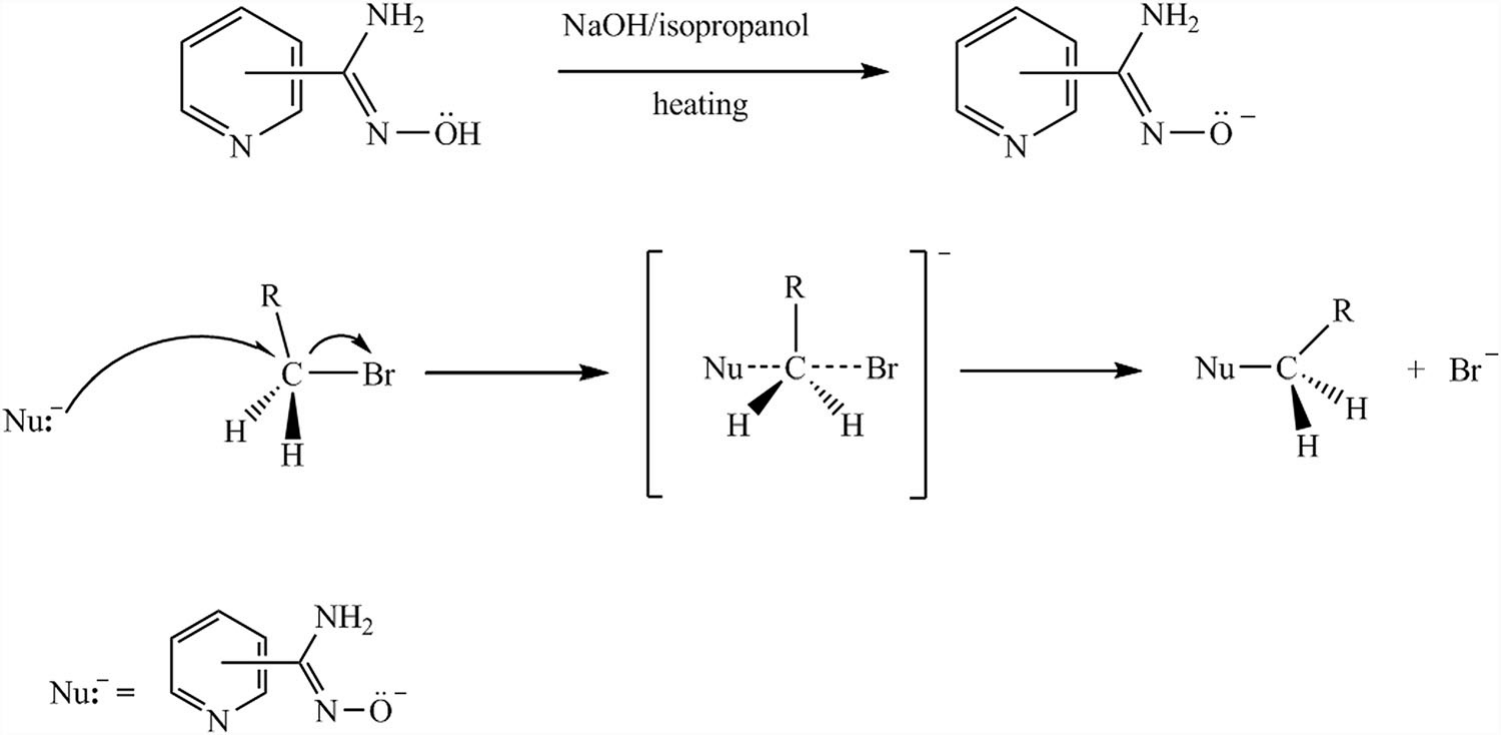
Mechanism of *O*-alkylation of *N*-hydroxypyridinecarboximidamide

*N*-substituted amidines, similar to well known simple amidoximes (Abele et al. 2003), have already been used as antimicrobial (Hall et al. 1998; Boykin et al. 1996), insecticidal (Paul 1981), herbicidal or plant growth regulatory agents (Farge et al. 1978, 1980) as well as DNA photo-cleavage agents (Karamtzioti et al. 2015) reactivators of nerve agent and pesticide poisoning (Kliachyna et al. 2014) and as regulator of blood pressure (Izawa et al. 1993). The most important key issues for the preparation of such compounds are a complexing nature of the *N*-hydroxypyridine-2- or -4-carboximidamide, which can coordinate to the divalent metal ions mainly through their pyridine or amine and imine nitrogen atom forming five- or six-membered chelate rings (Salonen et al. 2008; Salonen 2010; Konidaris et al. 2011; Coropceanu et al. 2014; Nandy et al. 2013). Preparation of simple *N*-hydroxyimidamides through an *O*-alkylation reaction has been described frequently (Izawa et al. 1993; Michaelis 1891; Eloy and Lenaers 1962). The alkylation is the process of introducing an alkyl group in the form of a carbocation, carbanion, or an alkyl radical into a molecule of an organic compound by a substitution reaction. In case of *N*-alkyloxypyridine-2- or -4-carboximidamide, the reaction mechanism consists of two stages (Fig. 1). In the first step sodium 1-amino-1-(2-, 3- or -4-pyridyl)imineoxide is formed by reaction of an appropriate *N*-hydroxypyridinecarboximidamide with strong base (NaOH, NaOR). The oxygen atom of the created organic sodium salts is thus highly activated and may act as a nucleophile. In the second step, the alkyl halide is attacked by the nucleophile according S_N_2 mechanism to form the final product*, N*-alkyloxypyridinecarboximidamide.

Elimination is always in competition with the substitution reaction and it may occur as unwanted side reactions. A common cause of the by-product formation, beyond alkyl halide structure, type of solvent, polarizability and basicity of nucleophile and type of leaving group, are a too long reaction time and too high temperature. Therefore, a quantitative analysis of the reaction enabling the choice of the optimal parameters is important for the design of an efficient method to synthesize hydrophobic *N*-alkyloxypyridinecarboximidamides containing decyloxy or 2-ethylhexyloxy group. The GC*/*MS method is the most commonly used method to determine the progress of N-substituted amidines synthesis (Mijin et al. 2004); however, the application of this method to analysis of compounds dissolved in water or a mixture of water with other diluent requires a modification of the simple preparation such as an extraction or diluents evaporation. Recent development and advancement in analytical technologies has emerged with more sophisticated hyphenated techniques. Among these, one of the most interesting is LC–MS/MS, which allows quantitatively analyze the different analytes in various matrices due to the inherent specificity and sensitivity. LC–MS/MS analyses depend on the use of triple quadrupole mass spectrometers operating in the MRM (multi reaction monitoring) mode (Bijlsma et al. 2009; Boleda et al. 2007; Petrovic et al. 2010). This mode of acquisition provides good sensitivity and selectivity and guarantees reliability of results by recording at least two or more specific SRM (selected reaction monitoring) transitions for each target analyte (Pozo et al. 2006; Jak et al. 2015). LC–MS/MS can be used for quantitative simultaneous analysis of organic compounds even from aqueous alcohol solution; therefore this instrument is the most frequently selected for environmental analysis.

The aim of the current study was to develop a simple and precise analytical method for the quantification of hydrophobic *N*-alkyloxypyridinecarboximidamides using LC–MS/MS technique directly from synthesis solutions.

## Experimental

### Chemicals and reagents

Methanol and acetonitrile of LC–MS grade were supplied from Sigma-Aldrich (Steinheim, Germany). Ammonium acetate used as a mobile phase additive was also purchased from Sigma-Aldrich (Steinheim, Germany). 2-pyridinecarbonitrile (98%), 3-pyridinecarbonitrile (98%), 4-pyridinecarbonitrile (98%), propan-2-ol, hydroxylamine hydrochloride (99%), 1-bromodecane and 1-bromo-2-ethylhexane were supplied from Sigma-Aldrich (Steinheim, Germany). NaOH (p.a), were supplied from POCH S.A. (Gliwice, Poland). The LC-grade water (below 1 μS/mL) was prepared by reverse osmosis in a Demiwa 5ROI system from Watek (Ledec nad Sazavou, Czech Republic), followed by double distillation from a quartz apparatus. Only freshly distilled water was used. Standards of *N*-(2-ethylhexyloxy)pyridine-2-carboximidamide, *N*-(2-ethylhexyloxy)pyridine-3-carboximidamide, *N*-(2-ethylhexyloxy)pyridine-4-carboximidamide, *N*-decyloxypyridine-2-carboximidamide, *N*-decyloxypyridine-3-carboximidamide and *N*-decyloxypyridine-4-carboximidamide were synthesised and confirmed by NMR spectroscopy (Bruker Avance II 400 MHz UltraShield Plus)

### Standards synthesis

Synthesis of the standards proceeded in a glass reactor with mechanical stirring at the boiling temperature of propan-2-ol. In the first stage, *N*-hydroxypyridine-2-, -3- and -4-carboximidamide prepared according to a procedure described by Bernasek (1956), as a solution (0.1 mol in 200 mL propan-2-ol) was heated with sodium hydroxide (0.12 mol in 50 mL water:propan-2-ol solution (2:8, v/v) for 30 min. Then, the mixture of 1-bromodecane or 1-bromo-2-ethylhexane (0.1 mol in 50 mL propan-2-ol) was added dropwise to this mixture, which includes *N*-hydroxypyridinecarboximidamide and NaOH, and next the whole mixture was heated at 85 °C for 3 h. Reaction products were purified by extraction with chloroform and finally by vacuum distillation. A purity of synthesized compounds was confirmed by NMR spectroscopy (Tables 1, 2).

**Table 1:** Structure and spectroscopic data of studied *N*-alkyloxypyridinecarboximidamides with 2-ethylhexyloxy group

*N*-(2-ethylhexyloxy)pyridine-2-carboximidamide (2-Eh)
^1^H NMR (400 MHz, CDCl_3_) *δ* (ppm): 0.91–0.96 (CH_3_, dd, 6*H*); 1.34–1.50 (CH_2_, m, 8*H*); 1.72–1.75 (CH, ttt, 1*H*); 4.04–4.06 (OCH_2_, d, 2*H*); 5.64 (NH_2_, s, 2*H*); 7.28–7.29 (H_py_, dd, 1*H*); 7.69–7.01 (H_py_, dd, 1*H*); 7.99–8.01 (H_py_, d, 1*H*); 8.54 (H_py_, d, 1*H*)
^13^C NMR (CDCl_3_) *δ* in ppm: 11.1; 14.1; 23.0; 23.9; 29.0; 29.1; 30.6; 39.1; 120.3; 124.2; 136.7; 136.7; 147.7; 149.3; 149.3
MS(ESI) *m/z* [M+H]^+^: 250.2

*N*-(2-ethylhexyloxy)pyridine-3-carboximidamide (3-Eh)
^1^H NMR (400 MHz, CDCl_3_) *δ* (ppm): 0.91–0.96 (CH_3_, dd, 6*H*); 1.34–1.50 (CH_2_, m, 8*H*); 1.72–1.75 (CH, ttt, 1*H*); 4.04–4.06 (OCH_2_, d, 2*H*); 5.64 (NH_2_, s, 2*H*); 7.54–7.55 (H_py_, dd, 1*H*); 7.60–7.61 (H_py_, d, 1*H*); 8.53–8.55 (H_py_, d, 1*H*); 8.85–8.83 (H_py_, s, 1*H*)
^13^C NMR (CDCl_3_) *δ* in ppm: 10.6; 13.6; 22.5; 23.4; 28.5; 30.0; 38.5; 119.9; 132.9; 146.5; 148.7; 149.9
MS(ESI) *m/z* [M+H]^+^: 250.2

*N*-(2-ethylhexyloxy)pyridine-3-carboximidamide (4-Eh)
^1^H NMR (400 MHz, CDCl_3_) *δ* (ppm): 0.91–0.95 (CH_3_, dd, 6*H*); 1.33–1.48 (CH_2_, m, 8*H*); 1.71–1.74 (CH; ttt, 1H); 4.06–4.08 (OCH_2_, d, 2*H*); 5.01 (NH_2_, s, 2*H*); 7.75-7.77 (H_py_, d, 2*H*); 8.65–8.66 (H_py_, d, 2*H*)
^13^C NMR (CDCl_3_) *δ* in ppm: 10.5; 13.6; 22.5; 23.4; 28.5; 30.0; 38.6; 119.4; 139.6; 148.5; 149.5
**MS(ESI)** *m/z* [M+H]^+^: 250.2

**Table 2:** Structure and spectroscopic data of studied *N*-alkyloxypyridinecarboximidamides with decyloxy group

*N*-decyloxypyridine-2-carboximidamide (2-D)
^1^H NMR (400 MHz, CDCl_3_) *δ* (ppm): 0.87–0.90 (CH_3_, t, 3*H*); 1.15–1.16 (CH_2_, kt, 2*H*); 1.28–1.43 (CH_2_, m, 12*H*); 1.70–1.77 (CH_2_, tt, 2*H*); 4.13–4.16 (OCH_2_, t, 2*H*); 5.75 (NH_2_, s, 2*H*); 7.34–7.37 (H_py_, dd, 1*H*); 7.75–7.79 (H_py_, dd, 1*H*); 8.08–8.10 (H_py_, d, 1*H*); 8.55–8.53 (H_py_, d, 1*H*)
^13^C NMR (CDCl_3_) *δ* in ppm: 13.7; 22.4; 25.6; 29.0; 29.1; 29.2; 29.5; 28.5; 30.6; 39.1; 120.3; 124.2; 136.7; 136.7; 147.7; 149.3; 149.3
MS(ESI) *m/z* [M+H]^+^: 278.2

*N*-decyloxypyridine-3-carboximidamide (3-D)
^1^H NMR (400 MHz, CDCl_3_) *δ* (ppm): 0.87–0.90 (CH_3_, t, 3*H*); 1.27 (CH_2_, m, 10*H*); 1.55–1.59 (CH_2_, tt, 2*H*); 1.70–1.74 (CH_2_, tt, 2*H*); 3.62–3.65 (CH_2_, tt, 2*H*); 4.11–4.15 (OCH_2_, t, 2*H*); 4.91 (NH_2_, s, 2*H*); 7.54–7.55 (H_py_, dd, 1*H*); 7.60–7.61 (H_py_, d, 1*H*); 8.63–8.65 (H_py_, d, 1*H*); 8.82–8.83 (H_py_, s, 1H)
^13^C NMR (CDCl_3_) *δ* in ppm: 13.9; 22.5; 25.8; 28.5; 29.1; 29.2; 29.3; 29.4; 30.0; 38.5; 119.9; 132.9; 146.5; 148.7; 149.9
MS(ESI) *m/z* [M+H]^+^: 278.2

*N*-decyloxypyridine-4-carboximidamide (4-D)
^1^H NMR (400 MHz, CDCl_3_) *δ* (ppm): 0.87–0.90 (CH_3_, t, 3*H*); 1.15–1.16 (CH_2_, kt, 2*H*); 1.28 (CH_2_, m, 10*H*); 1.41–1.43 (CH_2_, tt, 2*H*); 1.72–1.77 (CH_2_, tt, 2*H*); 4.13–4.16 (OCH_2_, t, 2*H*); 5.75 (NH_2_, s, 2*H*); 7.75–7.77 (H_py_, d, 2*H*); 8.55-8.56 (H_py_, d, 2*H*)
^13^C NMR (CDCl_3_) *δ* in ppm: 13.8; 22.4; 25.7; 28.5; 28.9; 29.0; 29.3; 29.5; 30.0; 38.6; 119.4; 139.6; 148.5; 149.5
MS(ESI) *m/z* [M+H]^+^: 278.2

### Equipments

Analyses were performed using the UltiMate 3000 RSLC LC system (Dionex, Sunnyvale, CA, USA) connected with an API 4000 QTRAP triple quadruple mass spectrometer (AB Sciex, Foster City, CA, USA). Chromatographic separation were done using reverse phase elution with a Spherisorb ODS2 column (50 mm × 4.6 mm I.D.: particle size 5 μm) (Waters, USA). The mass spectrometer was equipped with an electrospray interface operating in positive-ion mode.

Determination of *N-alkyloxypyridinecarboximidamines* at real synthesis conditions was done using a workstation EasyMax 102 Advanced laboratory reactor with a capacity of 100 mL. The reactor was equipped with reflux cooler, magnetic stirrer bar and temperature sensor. Precise temperature control (±0.1 °C) in the reactor was made possible by solid state thermostat.

### Operating conditions

#### LC–MS/MS analysis

The mobile phase used for the sample analysis consisted of 5 mmol L^−1^ ammonium acetate in a water and methanol mixture at flow rate of 0.6 mL min^−1^. The gradient was starting at 20% water and 80% methanol and changing linearly to 100% methanol in 2 min, with a final 4.5-min holding period. The duration between subsequent injections was 10 min.

ESI condition: curtain gas 10 psi, nebulizer gas 40 psi, auxiliary gas 40 psi, temperature 400 °C, ion spray voltage 5500 V and collision gas set to medium. Quantifications were performed in multiple reaction monitoring mode (MRM), and the following MRM transitions of [M+H]^+^ precursor ions → product ions were selected for each analyte: *N*-(2-ethylhexyloxy)pyridine-2-carboximidamine (*t*_R_ = 2.09 min)—*m/z* 250 → 105 (CE = 39 V), 250 → 120 (CE = 25 V) and 250 → 138 (CE = 23 V); *N*-(2-ethylhexyloxy)pyridine-3-carboximidamine (*t*_R_ = 1.61 min)—*m/z* 250 → 79 (CE = 61 V), 250 → 105 (CE = 41 V) and 250 → 121 (CE = 29 V); *N*-(2-ethylhexyloxy)pyridine-4-carboximidamine (*t*_R_ = 1.70 min)—*m/z* 250 → 79 (CE = 49 V), 250 → 121 (CE = 33 V) and 250 → 138 (CE = 27 V); *N*-decyloxypyridine-2-carboximidamide (*t*_R_ = 2.57 min)—*m/z* 278 → 78 (CE = 59 V), 278 → 96 (CE = 43 V) and 278 → 120 (CE = 27 V); *N*-decyloxypyridine-3-carboximidamide (*t*_R_ = 1.90 min)—*m/z* 278 → 78 (CE = 59 V), 278 → 96 (CE = 43 V) and 278 → 120 (CE = 27 V); *N*-decyloxypyridine-4-carboximidamide (*t*_R_ = 1.98 min.)—*m/z* 278 → 79 (CE = 51 V), 278 → 105 (CE = 59 V), 278 → 120 (CE = 33 V) and 278 → 121 (CE = 33 V). Collision energy (CE) was optimized with the “quantitative optimization” function of analyst 1.3.1 or 1.3.2. The monitored fragmentations were selected according to fragmentation pathways of pyridine amidoxime ethers described by Pearse and Jacobsson (1980). The dwell time for mass transition detected the MS/MS multiple reaction monitoring mode (MRM) was set at 50 ms.

#### Method validation

Linearity of the calibration was confirmed by analyzing solutions of standards (*N*-(2-ethylhexyloxy)pyridine-2-carboximidamide, *N*-(2-ethylhexyloxy)pyridine-3-carboximidamide, *N*-(2-ethylhexyloxy)pyridine-4-carboximidamide, *N*-decyloxypyridine-2-carboximidamide, *N*-decyloxypyridine-3-carboximidamine and *N*-decyloxypyridine-4-carboximidamide) at different concentrations ranging from 2.5 × 10^−5^ to 1.0 μg mL^−1^ [number of points = 16 (*n* = 3)]. Another determined value was limit of detection (LOD), defined as the concentration that yielded signal-to-noise (S/N) ratios greater than or equal to 3, and limits of quantification (LOQ), defined as the concentration of analyte yielding S/N ratios greater than or equal to 10. Accuracy and precision were carried out with three replicates of three different concentrations low, medium and high quality control samples ranging from 0.2 to 50 ng mL^−1^ and prepared mixtures containing also substrates of the reaction. Accuracy and precision was determined by injecting a sample with known concentration and calculation of the concentration from the graph and percentage relative standard deviation.

#### Application of method to monitor *N*-alkyloxypyridinecarboximidamide at real synthesis conditions

To perform the quantitative analysis of hydrophobic *N*-alkyloxypyridinecarboximidamides concentration at real synthesis conditions, a series of experiments were carried out under precise conditions. The reactions are done through a workstation EasyMax 102 Advanced laboratory reactor with a capacity of 100 mL. In reaction of synthesis, 1.37 g (0.01 mol) of starting substrate (*N*-hydroxypyridine-2-, -3- and -4-carboximidamide) dissolved in 100 mL of propan-2-ol was used. Stirring in the reactor was carried out using the magnetic stirrer at 500 rpm and the temperature of reactor was 50 °C. In a further step, 0.40 g (0.01 mol) of sodium hydroxide was added to the reaction mixture. The reaction was run for 15 min with a noticeable change of reaction mixture color to bright yellow, which indicated the occurrence of the reaction and the formation of the sodium salt of the *N*-hydoxypyridinecarboximidamide. The blank sample was taken before the addition of alkyl bromide (decyl or 2-ethylhexyl bromide) and dissolved in 5 mL of propan-2-ol. In the next step, 0.01 mol of alkyl bromide was added and the mixture was maintained for 120 min at 50 or 80 °C. Samples were taken every 5 min throughout the reaction. Before LC/MS/MS analysis all obtained samples were diluted to the maximum *N*-alkyloxypyridinecarboximidamide concentration 10 ng mL^−1^ and next were diluted with 5 mmol L^−1^ ammonium acetate to obtain a final analyte concentration of 0.25–0.5 ng mL^−1^. The concentration of the synthesized *N*-alkyloxypyridinecarboximidamide was calculated from the constructed linear regression equations, and additionally, standard was run before and after stock samples including one control sample and blank control sample.

## Results and discussion

### Methods development

Calibration curves for the analyte were constructed by plotting the peak area ratio versus analyte concentration. The regression equations, determination coefficients and linearity ranges of *N*-(2-ethylhexyloxy)pyridine-2-carboximidamide, *N*-(2-ethylhexyloxy)pyridine-3-carboximidamide and *N*-(2-ethylhexyloxy)pyridine-4-carboximidamide were shown in Table 3. All of the *N*-(2-ethylhexyloxy)pyridinecarboximidamides showed good linearity with regression coefficients >0.999. The LODs ranged from 0.013 to 0.025 ng mL^−1^ for *N*-(2-ethylhexyloxy)pyridine-3-carboximidamide, *N*-(2-ethylhexyloxy)pyridine-2-carboximidamide and *N*-(2-ethylhexyloxy)pyridine-4-carboximidamide, while the LOQ ranged from 0.1 ng mL^−1^ for *N*-(2-ethylhexyloxy)pyridine-4-carboximidamide to 0.25 ng mL^−1^ for *N*-(2-ethylhexyloxy)pyridine-2-carboximidamide and *N*-(2-ethylhexyloxy)pyridine-3-carboximidamide.

**Table 3:** Regression equations, linear ranges, LOD and LOQ parameters determined for *N*-(2-ethylhexyloxy)pyridine-2-carboximidamide (2-Eh), *N*-(2-ethylhexyloxy)pyridine-3-carboximidamide (3-Eh) and *N*-(2-ethylhexyloxy)pyridine-4-carboximidamide (4-Eh)

Analyte	Mass of degradation products	Linear range (ng mL^−1^)	Linear regression equation	LOD (ng mL^−1^)	LOQ (ng mL^−1^)
2-Eh	250 → 105	0.25–50	*y* = 3 × 10^7^ *x* + 22,744	0.013	0.25
	250 → 120	0.25–50	*y* = 5 × 10^7^ *x* + 33,964	0.013	0.25
	250 → 138	0.25–50	*y* = 5 × 10^7^ *x* + 36,829	0.013	0.25
3-Eh	250 → 79	0.25–500	*y* = 8 × 10^6^ *x* + 22,523	0.050	0.25
	250 → 105	0.25–250	*y* = 7 × 10^7^ *x* + 108,505	0.025	0.25
	250 → 121	0.25–500	*y* = 2 × 10^7^ *x* − 2381	0.025	0.25
4-Eh	250 → 79	0.10–500	*y* = 4 × 10^7^ *x* + 18,925	0.025	0.10
	250 → 121	0.10–100	*y* = 4 × 10^7^ *x* + 79,780	0.050	0.10
	250 → 138	0.10–250	*y* = 3 × 10^7^ *x* + 24,945	0.050	0.10

*r* ^2^ > 0.999; *n* = 3

In case of the *N*-decyloxypyridine-2-, -3- and -4-carboximidamide, the LOD was found to be 0.025 and 0.05 ng mL^−1^ for *N*-decyloxypyridine-2-carboximidamide and *N*-decyloxypyridine-3-carboximidamide, respectively (Table 4). The LOD of *N*-decyloxypyridine-4-carboximidamide depended on the MRM transitions and for transition *m/z* 278 → 79 the LOD was 0.05 ng mL^−1^, but for transition *m/z* 278 → 105, 278 → 120 and 278 → 121 the LOD was 0.025 ng mL^−1^. The LOQ also depended on MRM transitions and for *N*-decyloxypyridine-2-carboximidamide was ranged from 0.01 to 0.1 ng mL^−1^, but for *N*-decyloxypyridine-2-carboximidamide and *N*-decyloxypyridine-4-carboximidamide was ranged from 0.25 to 0.5 ng mL^−1^. The obtained calibration curves also showed excellent linearity with *r* ^2^ = 0.998–0.999 (Table 4).

**Table 4:** Regression equations, linear ranges, LOD and LOQ parameters determined for *N*-decyloxypyridine-2-carboximidamide (2-D), *N*-decyloxypyridine-3-carboximidamide (3-D) and *N*-decyloxypyridine-4-carboximidamide (4-D)

Analyte	Mass of degradation products	Linear range (ng mL^−1^)	Linear regression equation	LOD (ng mL^−1^)	LOQ (ng mL^−1^)
2-D	278 → 78	0.01–250	*y* = 6 × 10^7^ *x* + 5213	0.025	0.01
	278 → 96	0.10–250	*y* = 5 × 10^7^ *x* + 13,377	0.025	0.10
	278 → 120	0.10–250	*y* = 1 × 10^8^ *x* + 183,208	0.025	0.10
3-D	278 → 96	0.50–500	*y* = 1.8 × 10^5^ *x* − 346	0.050	0.50
	278 → 105	0.25–500	*y* = 4 × 10^7^ *x* + 2623	0.050	0.25
	278 → 120	0.30–10	*y* = 8.7 × 10^5^ *x* − 149	0.050	0.30
4-D	278 → 79	0.50–250	*y* = 5 × 10^7^ *x* + 100,240	0.025	0.50
	278 → 105	0.25–250	*y* = 2 × 10^7^ *x* + 35,887	0.050	0.25
	278 → 120	0.50–1000	*y* = 3 × 10^6^ *x* + 3438	0.050	0.50
	278 → 121	0.50–250	*y* = 3 × 10^7^ *x* + 41,094	0.050	0.50

*r* ^2^ > 0.999; *n* = 3

### Accuracy and precision of analytical method was also calculated

The accuracy of developed method was studied using single solutions at concentration levels ranging from 0.2 to 50 ng mL^−1^ and prepared mixtures containing also substrates of the reaction. The samples of known concentration were injected into the LC/MS/MS system. The peak area was used for calculating the *N*-alkyloxypyridinecarboximidamides concentrations using the corresponding regression equations (Tables 3, 4). Accuracy percentages were calculated. The method was found accurate for all studied compounds with average recovery of 97.72 ± 1.38, 100.81 ± 0.93, 99.03 ± 1.29, 96.99 ± 1.39, 98.89 ± 0.89 and 101.06 ± 1.12% for 2-Eh, 3-Eh, 4-Eh, 2-D, 3-D and 4-D, respectively.

Precision was carried out by analyzing laboratory prepared mixtures of appropriate *N*-alkyloxypyridinecarboximidamide, alkyl bromide, *N*-hydroxypyridinecarboximidamide, NaOH, ethanol, water within the linearity range (Tables 3, 4) on the same day (*n* = 3) and on three consecutive days using the same procedure. The accuracy of the method for the selected concentrations was calculated using the corresponding regression equations and was found to be satisfactory. The percentage relative standard deviation values for 2-Eh and 2-D were less than 1.5%, and for 3-Eh, 4-Eh, 3-D and 4-D were less than 1.0%.

### *N*-alkyloxypyridinecarboximidamides determination at real synthesis conditions

**Fig. 2:**
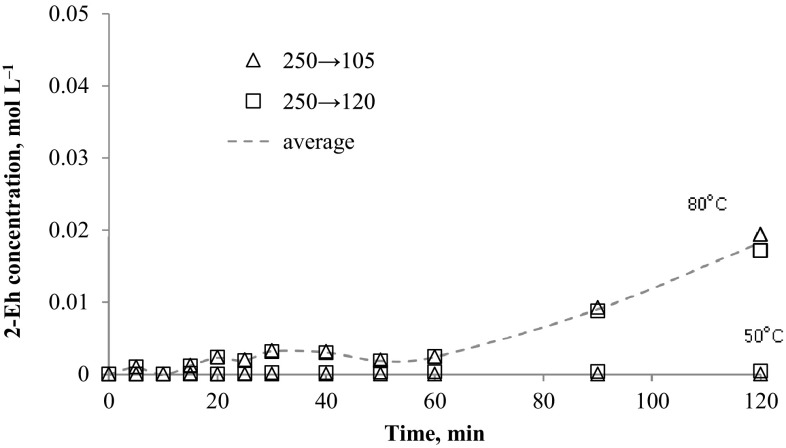
Changes in concentration of *N*-(2-ethylhexyloxy)pyridine-2-carboximidamide during reaction carried out at 50 and 80 °C

**Fig. 3:**
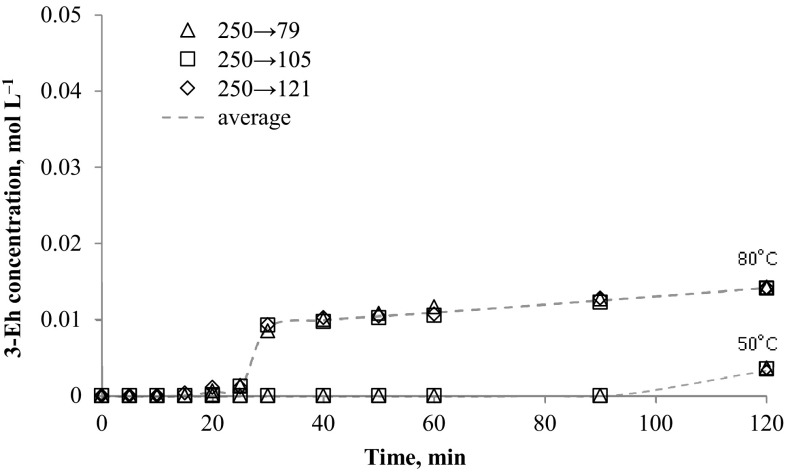
Changes in concentration of *N*-(2-ethylhexyloxy)pyridine-3-carboximidamide during reaction carried out at 50 and 80 °C

**Fig. 4:**
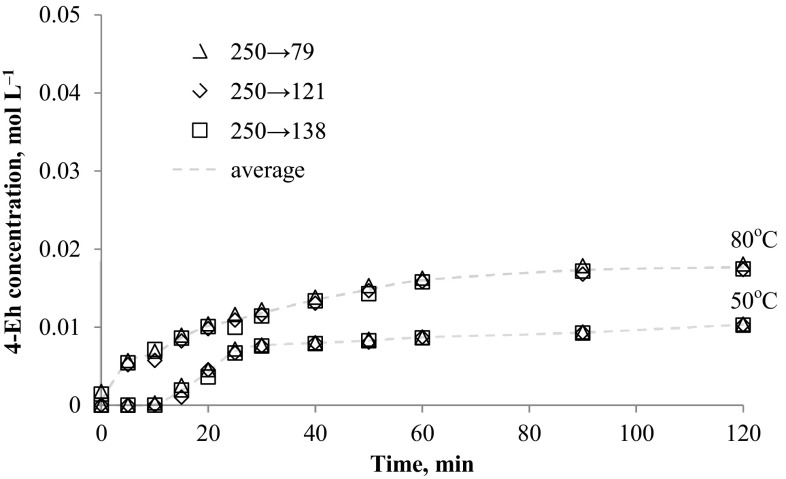
Changes in concentration of *N*-(2-ethylhexyloxy)pyridine-4-carboximidamide during reaction carried out at 50 and 80 °C

**Fig. 5:**
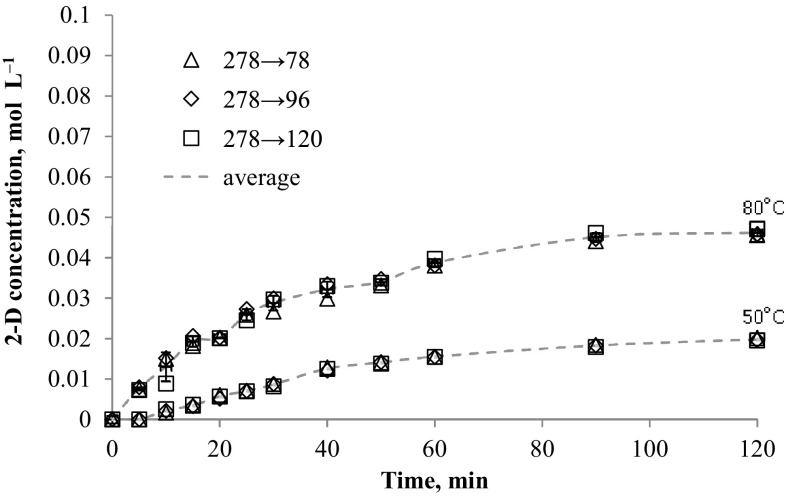
Changes in concentration of *N*-decyloxypyridine-2-carboximidamide during reaction carried out at 50 and 80 °C

**Fig. 6:**
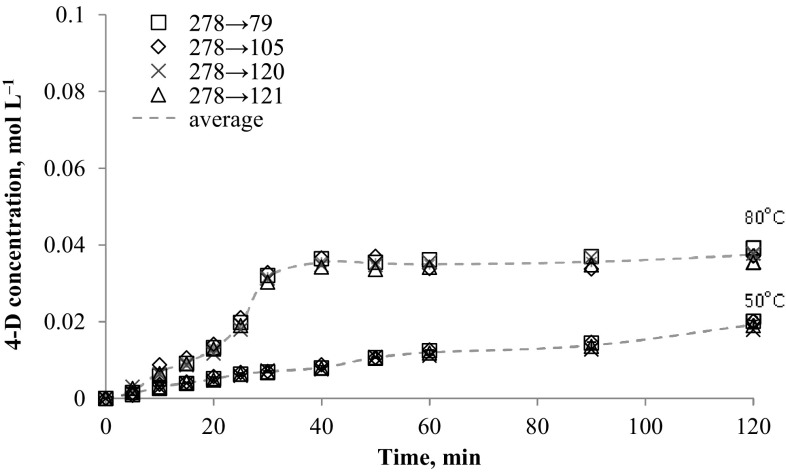
Changes in concentration of *N*-decyloxypyridine-4-carboximidamide during reaction carried out at 50 and 80 °C

**Fig. 7:**
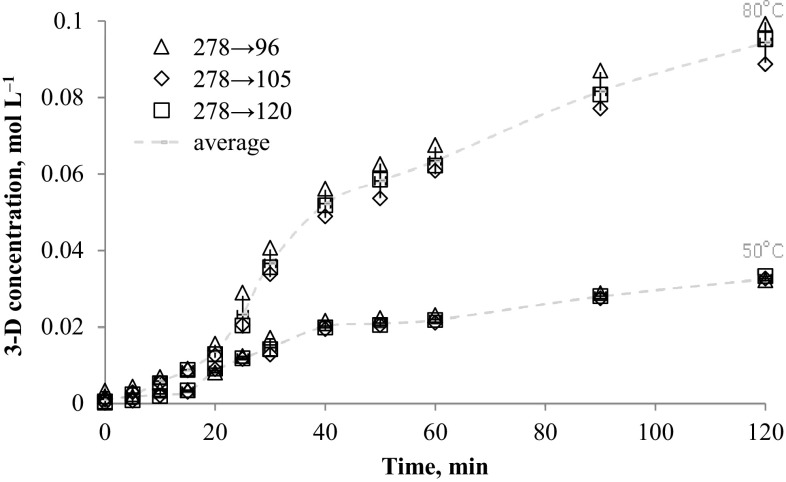
Changes in concentration of *N*-decyloxypyridine-3-carboximidamide during reaction carried out at 50 and 80 °C

The method was applied to determination of *N*-alkyloxypyridinecarboximidamides directly from reaction mixture (water-propan-2-ol mixture with 0.1 mol NaOH). The data presented in Figs. 2, 3, 4, 5, 6 and 7 indicates the influence of an alkyl bromide structure, reaction time and temperature of the reaction on the *O*-alkylation efficiency. It was found that the straight chain alkyl bromide reacted most rapidly than branched alkyl bromide, and as expected, the enhancement of the reaction rate strongly depends on reaction temperature. The *O*-alkylation reaction with 2-ethylhexyl bromide is slightly observed at 50 °C. Especially in case of sodium 1-amino-1-(2-pyridyl)imineoxide and sodium 1-amino-1-(3-pyridyl)imineoxide, even a 2-h reaction generated less than 1% of the final product (Figs. 2, 3). Sodium 1-amino-1-(4-pyridyl)imineoxide reacted efficiently with 2-ethylhexyl bromide, but the yield of the *O*-alkylation was still poor (10.00 ± 0.1%) (Fig. 4). It seems that the *O*-alkylation with 2-ethylhexyl bromide requires to proceed at higher temperature, as confirmed by the reaction of sodium 1-amino-1-(2-pyridyl)imineoxide with 2-ethylhexyl bromide at 80 °C (Fig. 4). 2-ethylhexyl bromide also reacted more efficiently with sodium 1-amino-1-(3-pyridyl)imineoxide and sodium 1-amino-1-(4-pyridyl)imineoxide giving *N*-(2-ethylhexyloxy)pyridine-3-carboximidamide and *N*-(2-ethylhexyloxy)pyridine-4-carboximidamide with yield of 14.18 ± 0.4 and 17.63 ± 0.4%, respectively (Figs. 3, 4).

In case of decyl bromide, the reaction with sodium 1-amino-1-(2-pyridyl)imineoxide and sodium 1-amino-1-(4-pyridyl)imineoxide at a higher reactor temperature also causes more efficient substitution reaction leading to generate final product in 46.10% (±0.82) 2-D and in 37.36% (±1.53) 4-D (Figs. 5, 6). The high-efficiency synthesis was achieved for the mixture of sodium 1-amino-1-(3-pyridyl)imineoxide and decyl bromide. For this mixture at 50 °C *N*-decyloxypyridine-3-carboximidamide was generated in 32.66% (±0.57), but the more intensive heating enabled a complete conversion of the substrates to the desired product (Fig. 7).

## Conclusions

The LC–MS/MS method to determine *N*-(2-ethylhexyloxy)pyridine-2-carboximidamide, *N*-(2-ethylhexyloxy)pyridine-3-carboximidamide, *N*-(2-ethylhexyloxy)pyridine-4-carboximidamide, *N*-decyloxypyridine-2-carboximidamide, *N*-decyloxypyridine-3-carboximidamide and *N*-decyloxypyridine-4-carboximidamide was developed and validated in terms of linearity, precision and accuracy. The developed method is an accurate and easily performed method for determining amphiphilic *N*-alkyloxypyridinecarboximidamides directly from reaction mixture. The method was relatively unsusceptible to matrix effects, especially to the presence of reaction substrates. The limits of detections were at low levels (0.0125–0.05 ng mL^−1^) and precision was below 1.5%. The LC–MS/MS enables the identification, detection and quantitation of the *N*-alkyloxypyridinecarboximidamides at low concentration at real synthesis conditions.

The LC–MS/MS *N*-alkyloxypyridinecarboximidamides determination at the real synthesis conditions enables monitoring of *O*-alkylation reaction progress. The data provided indicated that regardless on the alkyl bromide structure and position of aminoimineoxide moiety at pyridine ring, the intensive heating enabled a more efficient conversion of the substrates to the desired product.

The conducted studies (method validation and verification at real synthesis conditions) enable to select the MRM transition, which guarantee precise value of concentration of the synthesized *N*-alkyloxypyridinecarboximidamide. The selected MRM transitions (*m/z*) are 250 → 120 (2-Eh), 250 → 105 (3-Eh), 250 → 79 (4-Eh), 278 → 96 (2-D), 278 → 105 (3-D) and 278 → 120 (4-D).
